# Supplementary material for: Developing and validating an individualized breast cancer risk prediction model for women attending breast cancer screening
Source: PLoS One. 2021 Mar 23;16(3):e0248930. doi: 10.1371/journal.pone.0248930 (PMC7987139; doi:10.1371/journal.pone.0248930)
Supplement: Attachment — Submitted filename: Response letter.docx [file pone.0248930.s002.docx]

Comments from the Editors and Reviewers:

6. It is a shame that the data cannot be made available due to confidentiality. There are precedents for researchers releasing such data used to fit risk models. For example, you can access a modified version of the BCSC data used for their model, where categories have been coded (e.g. not individual year of age). I'd encourage the authors to consider trying to do this if at all possible. What are the confidentiality issues here? It would also be worth making your code available, for transparency of statistical methods used.

Response:

We thank the reviewer for this contribution. We have uploaded the database to the Harvard Dataverse online repository.

The data is accessible with DOI: https://doi.org/10.7910/DVN/3T7HCH

- Fantastic thank you. Would it also be possible to make available the analysis code used for this paper - for complete reproducibility?

**Response:** We thank the reviewer for this contribution. Yes, it is possible and we agree it will help for understanding and reproducibility of the paper. We have uploaded the code publicly to github at the following address:

<https://github.com/JlouroA/IRISModelCode>

We have split the code in two parts, a small part in SPSS where we split the database in the estimation and the validation subcohort and the R code where all the analyses of the paper are done (both model development and validation).

**Note to the reviewer:** For a better understanding of the article, we have modified the term "model subcohort" to "estimation subcohort" both in the manuscript and in this response letter to refer to the part of the cohort used for the model development. We believe that speaking of "estimation cohort" and "validation cohort" is much clearer.

12. What robust confidence intervals (method).

Response:

In particular, we used the robust standard error reported by the Huber sandwich estimator to create the robust confidence intervals. This is a standard estimation method to obtain robust estimates and is the one reported both by the Standard Cox function (coxph function of the R package survival) and by the model used for the Partly Conditional Cox model (partlyconditional package).

More information of this package can be found at:

https://github.com/mdbrown/partlyconditional

- Did you include this in the text?

**Response:** Thank you for this suggestion. This was not included in the text and we agree it can improve the clearance of the paper. We have added a new sentence and new reference in the methods section of the article to state the method used to estimate the confidence intervals.

**New text in methods (underlined):** Robust standard errors were used to estimate 95% confidence intervals using the Huber sandwich estimator [26].

26.	Freedman, DA. On the So-Called ‘Huber Sandwich Estimator’ and ‘Robust Standard Errors.’ The American Statistician, vol. 60, no. 4, 2006, pp. 299–302. JSTOR, www.jstor.org/stable/27643806. Accessed 23 Feb. 2021.

14. What were reasons for censoring? How many for each reason. e.g. Did anyone die? What if a woman did not attend her screening visit? What if she was older than 69y?

Response:

We thank the reviewer for this comment. As we extracted our data directly from the population-based screening program databases we are assured of having the maximum follow-up of these women. We collected in a comprehensive and systematic way all the participations that these women have made in the breast cancer screening program.

However, we cannot know exactly what the cause of each loss of follow-up is. As we do all the analysis by screening participation and not by women, and taking into account the time between mammograms, there is not any problem at all for the analysis if a woman skips one of the screening tests but she returns to the screening later.

Of the 121,969 women in our cohort, 63,694 had their last mammogram within the last two years of the study, which are 2014 and 2015. These women were censored at the end of the study period. 20,436 women of the remaining had their last mammogram at age 68 or 69, so these women completed their screening process during the study follow-up.

Of the remaining 37,839 we know that the majority are women who have decided not to participate in the 2014-2015 round or who have changed health areas and therefore are not in our study population. Regarding the women who die, the screening program does not have an exhaustive record of this cause and they appear to us as non-participating women.

- Did you include this in the text?

**Response:**

We thank the reviewer for this contribution. We agree that this information could be included in the text. We have added a new paragraph in the discussion to make it clearer.

**New text in discussion (underlined):** Another limitation might be the reason for censoring. Over 52% of women in the cohort had their last mammogram in the last two years of the study follow-up and 17% of women had their last mammogram at ages 68 or 69 years. Most of the remaining 31% are women who did not participate in the 2014-2015 round or who have changed health areas and thus are not in our study population. The screening program does not have an exhaustive record of which women die and, therefore, we cannot differentiate them from non-participating women.

15. How did you model age? In piecewise constant 5y intervals? Why?

Response:

To build the model we used age as a quantitative variable. By using a partly conditional Cox model we performed the analysis by screening participation instead of by women. Therefore, risk factors through time were incorporated as time-dependent covariates, age included.

However, to present the results in an easy way we showed age in piecewise constant 5-year intervals in tables. We believe this is a commonly used way to present results (e.g. BCSC, see Tice JA, Miglioretti DL, Li CS et al. Breast Density and Benign Breast Disease: Risk Assessment to Identify Women at High Risk of Breast Cancer. J Clin Oncol. 2015;33(28):3137-43)

- Did you include in the text? (If you made your analysis code available this would be even more transparent)

**Response:** We have added a new sentence in the text to facilitate the understanding of readers. Thank you for this good suggestion.

**New text in methods (underlined):** We estimated the age-adjusted hazard ratios (aHR) and the 95% confidence intervals (95%CI) for the breast cancer incidence for each category of family history, previous BBD, and previous mammographic features. Age was included in the model as a continuous variable.

16. Did you AUC consider follow-up time? e.g. Some will have entered cohort later than others. It appears that you look at 2y risk as predictor and yes/ no cancer in that period. Multiple values for each person. Did you adjust for loss of independence due to this? Standard Hanley and McNeil would not?

Response:

We thank the reviewer for this interesting comment. Yes, we do consider follow-up time at every horizon for every individual in the cohort. In table 3 (previous table 4), we estimated the AUC for every 2-year interval, which means, that we estimated the 20-year risk AUC for those individuals followed for 20 years, the 18-year risk AUC for those followed for at least 18 years, the 16-year risk AUC for those followed for at least 16 years, and so on. The AUC for each time horizon was estimated with all the women in the validation cohort followed at least that time, using the predicted risk of the model and whether she has developed a tumor or not at the specific time horizon.

- Thank you. But is the risk score a time-varying covariate over the 20y horizon, or you use the baseline assessment? The two are not the same and good to clarify in paper methods.

**Response:** We thank the reviewer for this comment. This is an important question. We will try to clarify this point:

The AUC is estimated as a baseline assessment, although risk factors are time-changing variables. For example, the 18-year AUC is calculated by taking those women in the validation subcohort followed for at least 18 years and calculating their 18-year risk with their risk factors fixed at their baseline mammogram.

With the predicted risk and whether they developed a breast cancer or not, we can calculate the AUC of that time horizon (the 18-year risk in this example).

We apply this calculation for all time horizons independently (2-year, 4-year,…,20-year).

We agree that this methodological aspect can be misleading. We have added a new sentence in the text to try to make it clearer for the reader.

**New text in methods (underlined):**

The discriminatory accuracy of our model was assessed by estimating the area under the receiving operating characteristic curve (AUC) for each 2-year interval based on the predicted risks for each woman and ~~each woman’s final outcome~~ whether she developed breast cancer during the time interval or not [29]. The predicted risks were calculated using the model coefficient estimates at the baseline mammogram for those women in the validation cohort who have been followed for a time greater than or equal to the time horizon being estimated. The AUC measured the ability of the model to discriminate between women who will develop breast cancer from those who will not.

25. Table 4. I don't think you give sufficient detail for me to know how you calculated this table (methods). In particular, how did you estimate expected risk? Is it based on updating risk factors through time? Please provide enough detail in the methods for reproducibility.

Response: We thank the reviewer for this important observation. We agree with this comment, both in table 3 and 4, we must explain how we estimate the expected risk. We have tried to clarify it better by adding the following sentence to methods.

New text methods (underlined): The expected breast cancer rate was calculated as the average of the model predicted risk for each woman in a specific subgroup.

- Can you say more about this? Predicted risk of breast cancer to what time? There are different ways to do this, cf. I assume cumulative hazard to time each measurement of predictors / event / censoring, but useful to clarify.

**Response:** We thank the reviewer for this comment. We agree that this methodology is complex and it could be better explained. We will try to clarify:

In table 3 every time horizon uses a different estimate. ; 2-year risk estimate, 4-year risk estimate, etc…

Let’s take the 4-year risk estimate as an example:

The observed rate was estimated using the Kaplan Meier risk estimate at 4 years of the estimation subcohort.

The expected rate is calculated as the mean of the predictions of the 4-year risk estimates for all women in the validation subcohort.

All other time horizons on table 3 are calculated the same way.

However, to compute table 4 we used the 10-year risk estimates as a reference, but the table can be replicated for every time horizon. Using the 10-year estimates is just an illustrative example.

On table 4, we estimated the expected and observed ratio for each risk factor: With or without family history, with no BBD, with proliferative BBD, etc...

Similarly to table 3, the observed rate is the Kaplan Meier estimate at 10 years in the specific risk group of the estimation subcohort (Example, only those with family history). The expected breast cancer rate was calculated as the average of the 10-year risk estimates for each woman in the specific risk group of the validation subcohort (Example, only those with family history).

All code is now available in the aforementioned github repository.

We have added a new sentence in the text to try to make it clearer for the reader. Thanks for the suggestion.

**New text in methods (underlined):**

To assess calibration, we calculated the ratio between the expected breast cancer rate in the validation subcohort versus the observed rate in the ~~model~~ estimation subcohort. To ~~consider~~ account for censoring, the observed rate was estimated using ~~a~~ the Kaplan-Meier estimator. The expected breast cancer rate was calculated as the average of ~~the model predicted risk for each woman in a subgroup.~~ the risk estimates in the validation subcohort. The expected breast cancer rate in a specific risk group was calculated as the average of the risk estimates for each woman in that risk group of the validation subcohort. The expected-to-observed (E/O) ratio assessed whether the number of women predicted to develop breast cancer from the model matched the actual number of breast cancers diagnosed in the validation subcohort. An E/O ratio of 1.0 indicates perfect calibration.
